# Predictive factors for return to work after shoulder arthroplasty

**DOI:** 10.1016/j.jsea.2026.100058

**Published:** 2026-06-26

**Authors:** Willem Geuskens, Alexander Poelmans, Anthony Timmerman, Willem-Jan Metsemakers, Filip Verhaegen, Philippe Debeer

**Affiliations:** a Department of Orthopaedic Surgery, University Hospitals Leuven, Leuven, Belgium; b UPMC Sports Surgery Clinic, Dublin, Ireland; c Royal College of Surgeons in Ireland, Dublin, Ireland; d Department of Trauma Surgery, University Hospitals Leuven, Leuven, Belgium; e Department of Development and Regeneration, KU Leuven, Leuven, Belgium; f Department of Development and Regeneration, Institute for Orthopaedic Research and Training (IORT), KU Leuven, University Hospitals Leuven, Leuven, Belgium

**Keywords:** Shoulder arthroplasty, Return to work, Osteoarthritis, Cuff tear arthropathy, Occupational outcomes, Patient-reported outcome measures

## Abstract

**Background:**

Return to work (RTW) is a crucial outcome parameter for active patients undergoing shoulder arthroplasty. However, literature reporting on RTW rates is scarce. The purpose of this study was to assess the RTW rate following shoulder arthroplasty in patients of working age and to identify factors associated with successful RTW.

**Methods:**

This retrospective cohort study included patients under 63 years of age who underwent shoulder arthroplasty with a minimal follow-up of 2 years. Participants completed 2 types of questionnaires: a work-specific questionnaire and questionnaires to assess their shoulder function (the Patient-Derived Constant Murley Score, the Subjective Shoulder Value, Oxford Shoulder Score, the Simple Shoulder Test, and a Work-Related Questionnaire for Upper extremity disorders). Factors related to RTW were analyzed using the Mann-Whitney *U* test and chi-squared test.

**Results:**

Out of the 103 included patients, 69 (67.0%) returned to work, with 40 of them (58.0%) resuming their previous job full-time. The mean time to RTW was 16 weeks. Factors associated with successful RTW included higher educational level, less physically demanding work, pre-operative sick leave of less than one year, and post-operative support received at work. Age, gender, body mass index, American Society of Anesthesiologists score, type of procedure, indication for surgery, and post-operative complications did not significantly influence RTW rates. Patients who returned to work showed significantly better patient-reported outcome measures.

**Conclusion:**

Sixty-seven percent of patients successfully returned to work after shoulder arthroplasty. Predictive factors for successful RTW included educational level, job physicality, pre-operative sick leave, and the extent of post-operative support received at work. Neither the type of arthroplasty, indication for surgery, presence of complications nor the need for revision surgery significantly impacted RTW outcomes. These findings may guide pre-operative counseling and help to manage post-operative work-related expectations in patients undergoing shoulder arthroplasty.

Shoulder arthroplasty is an increasingly common procedure, performed to treat a variety of degenerative and traumatic conditions affecting the glenohumeral joint.^7^^,^^10^^,^^17^^,^^23^^,^^24^ Both anatomic total shoulder arthroplasty (aTSA) and reverse total shoulder arthroplasty (rTSA) have demonstrated good to excellent outcomes in terms of pain relief and functional improvement, particularly in older patients.^14^^,^^25^ However, as the indications for the procedure have expanded, younger and more active patients are now undergoing shoulder arthroplasty.^2^, ^3^, ^4^ In this population, return to work (RTW) is a crucial parameter of successful post-operative rehabilitation. The ability to return to work not only enhances the quality of life and psychological well-being but also carries substantial socioeconomic implications. Failure to return to work results in productivity loss and places additional strain on social security systems.^9^ Despite its importance, RTW outcomes after shoulder arthroplasty remain underreported.^11^^,^^19^

Current literature on RTW after shoulder arthroplasty is scarce and frequently lacks detailed information on work-related variables. A wide variety of RTW rates have been reported, yet many studies suffer from small sample sizes and fail to report on factors such as physical workload, educational level, or patient-reported outcome measures (PROMs), all of which are likely to impact RTW success.^11^^,^^19^ This gap in the literature limits surgeons' ability to properly counsel patients pre-operatively and manage post-operative patient expectations, particularly in a working-age population.

Therefore, the purpose of this study was to evaluate the RTW rate after shoulder arthroplasty in a working-age population and to identify potential predictive factors influencing successful RTW.

## Methods

### Patient selection

This retrospective multisurgeon, single-center study reviewed data from patients who underwent a shoulder arthroplasty procedure between January 2010 and December 2022. Inclusion criteria were adult patients younger than or equal to 63 years old at the time of surgery who underwent one of the following shoulder arthroplasty procedures: aTSA, rTSA, humeral hemiarthroplasty (HHA), or revision shoulder arthroplasty. The age of 63 years was selected because the minimum follow-up was 2 years and the statutory retirement age in Belgium was 65 years during the study period. This ensured that all included patients were theoretically still of working age at the minimum follow-up. Exclusion criteria included age over 63 years at the time of surgery, limited understanding of Dutch, patients who passed away during the follow-up period, a history of prolonged pre-operative sick leave due to nonshoulder-related long-term disability, and early retirement from work prior to surgery. Long-term disability, referred to as “invalidity” in the Belgian social security system, was defined as a formal status granted to individuals deemed unfit to work for an extended period due to medical reasons. Patients with invalidity directly related to the affected shoulder were not excluded. This study was approved by the institutional Research Ethics Committee.

### Data collection

Eligible patients were identified through the hospital's electronic database and subsequently contacted by phone. Patients who agreed to participate were sent study materials by mail, including a study information sheet, an informed consent form, and a set of questionnaires.

### Outcome measurements

Pre-operative patient demographics collected included age at the time of surgery, gender, dominant limb, body mass index (BMI), American Society of Anesthesiologists (ASA) score, smoking status, and surgical procedure.

All participants completed a set of questionnaires. The first questionnaire, a work-specific questionnaire, comprised 15 multiple-choice questions designed to assess time to return to work and work-related factors, including pre-operative sick leave, pre-operative type of employment, physically demanding work, satisfaction at work before surgery, post-operative support received at work, and educational level. Physically demanding work and post-operative support received at work were recorded according to the categories presented in Table I. Additional questionnaires included the Dutch versions of the Patient-Derived Constant Murley Score, the Subjective Shoulder Value, the Oxford Shoulder Score, the Simple Shoulder Test score, and the Work-Related Questionnaire for Upper Extremity Disorders.

**Table I:** Overview of the comparisons of variables between the RTW group and the no RTW group.

Variable	Unit	RTW	No RTW	Total	*P* value
Age, median (range)	Yr	54.00 (24.00-62.00)	55.00 (32.00-62.00)		.32∗
BMI, median (range)	kg/m^∧^2	28.70 (18.40-40.90)	27.80 (21.80-41.90)		.77∗
Gender, n (%)					
Female		24.00 (58.54%)	17.00 (41.46%)	41.00	.14†
Male		45.00 (72.58%)	17.00 (27.42%)	62.00	
Surgery on dominant shoulder?, n (%)					
Yes		44.00 (63.77%)	25.00 (36.23%)	69.00	.26†
No		24.00 (75.00%)	8.00 (25.00%)	32.00	
ASA score, n (%)					
1		13.00 (81.25%)	3.00 (18.75%)	16.00	.46†
2		46.00 (63.01%)	27.00 (36.99%)	73.00	
3		9.00 (75.00%)	3.00 (25.00%)	12.00	
4		1.00 (50.00%)	1.00 (50.00%)	2.00	
Pre-operative sick leave, n (%)					
0-2 weeks		29.00 (74.36%)	10.00 (25.64%)	39.00	**<.01**†
2-4 weeks		5.00 (100%)	0.00 (0.00%)	5.00	
1-3 mo		12.00 (85.71%)	2.00 (14.29%)	14.00	
3-6 mo		9.00 (75.00%)	3.00 (25.00%)	12.00	
6-12 mo		8.00 (80.00%)	2.00 (20.00%)	10.00	
>1 yr		6.00 (26.09%)	17.00 (73.91%)	23.00	
Physically demanding work, n (%)‡					
Very low demanding		21.00 (91.30%)	2.00 (8.70%)	23.00	**<.01**†
Low demanding		10.00 (90.91%)	1.00 (9.09%)	11.00	
Neutral		23.00 (76.67%)	7.00 (23.33%)	30.00	
High demanding		9.00 (42.86%)	12.00 (57.14%)	21.00	
Very high demanding		6.00 (33.33%)	12.00 (66.67%)	18.00	
Satisfaction at work before surgery, n (%)					
Very satisfied		23.00 (65.71%)	12.00 (34.29%)	35.00	.12†
Satisfied		29.00 (80.56%)	7.00 (19.44%)	36.00	
Unsatisfied		11.00 (52.38%)	10.00 (47.62%)	21.00	
Very unsatisfied		6.00 (54.55%)	5.00 (45.45%)	11.00	
Post-operative support received at work, n (%)§					
Yes		25.00 (86.21%)	4.00 (13.79%)	29.00	**.04**†
Little		15.00 (83.33%)	3.00 (16.67%)	18.00	
None		28.00 (62.22%)	17.00 (37.78%)	45.00	
Pre-operative type of employment, n (%)§					
Civil servant		15.00 (88.24%)	2.00 (11.76%)	17.00	.16†
No employment		1.00 (50.00%)	1.00 (50.00%)	2.00	
Salaried		46.00 (73.02%)	17.00 (26.98%)	63.00	
Self-employed		5.00 (50.00%)	5.00 (50.00%)	10.00	
Use of tobacco at the time of surgery, n (%)					
Active		14.00 (60.87%)	9.00 (39.13%)	23.00	.78†
Never		29.00 (69.05%)	13.00 (30.95%)	42.00	
Former		26.00 (68.42%)	12.00 (31.58%)	38.00	
Social status, n (%)					
Living alone		5.00 (62.50%)	3.00 (37.50%)	8.00	.93†
Married		43.00 (67.19%)	21.00 (32.81%)	64.00	
Divorced		11.00 (73.33%)	4.00 (26.67%)	15.00	
Living together		8.00 (66.67%)	4.00 (33.33%)	12.00	
Widow		2.00 (50.00%)	2.00 (50.00%)	4.00	
Educational level, n (%)					
College/university		32.00 (82.05%)	7.00 (17.95%)	39.00	**<.01**†
Secondary school		33.00 (63.46%)	19.00 (36.54%)	52.00	
No degree		4.00 (33.33%)	8.00 (66.67%)	12.00	
Type of arthroplasty, n (%)					
Humeral hemiarthroplasty		5.00 (83.33%)	1.00 (16.67%)	6.00	.46†
Reverse shoulder arthroplasty		27.00 (61.36%)	17.00 (38.64%)	44.00	
Total shoulder arthroplasty		37.00 (69.81%)	16.00 (30.19%)	53.00	
Indication for surgery, n (%)					
Arthrosis		40.00 (71.43%)	16.00 (28.57%)	56.00	.85†
Cuff arthropathy		9.00 (64.29%)	5.00 (35.71%)	14.00	
Fracture		6.00 (60.00%)	4.00 (40.00%)	10.00	
Other		5.00 (55.56%)	4.00 (44.44%)	9.00	
Revision surgery		9.00 (64.29%)	5.00 (35.71%)	14.00	
Post-operative complication?, n (%)					
Yes		2.00 (50%)	2.00 (50%)	4.00	.46†
No		67.00 (67.68%)	32.00 (32.32%)	99.00	
Revision surgery occurred?, n (%)					
Yes		6.00 (54.55%)	5.00 (45.45%)	11.00	.35†
No		63.00 (68.48%)	29.00 (31.52%)	92.00	
SSV, median (range)		80.00 (1.00-100.00)	55.00 (10.00-85.00)		**<.01**∗
OSS score, median (range)		43.00 (14.00-48.00)	26.00 (7.00-46.00)		**<.01**∗
SST score, median (range)		75.00 (0.00-100.00)	33.30 (0.00-100.00)		**<.01**∗
WORQ-UP score, median (range)		23.00 (13.00-79.00)	45.50 (17.00-80.00)		**<.01**∗

*BMI*, body mass index; *SSV*, Subjective Shoulder Value; *OSS*, Oxford Shoulder Score; *SST*, Simple Shoulder Test; *WORQ-UP*, Work-Related Questionnaire for Upper extremity disorders; *ASA*, American Society of Anesthesiologists.

Values are presented as median (range) or n (%). Percentages represent row percentages, indicating the proportion of patients within each category who returned to work (RTW) or did not return to work (no RTW).

A *P* value <.05 was considered statistically significant.

∗ Mann-Whitney *U* test.

† Chi-squared test.

‡ Job physicality categories are defined among the intensity of the following descriptions: 1. Very low demanding: office work (typing, computer work, phone handling…). 2. Low demanding: manual work performed at a table. 3. Neutral: standing work. 4. High demanding: pushing and pulling wheelbarrows. 5. Very high demanding: climbing ladders and stairs.

§ Patients with pre-operative long-term disability (invalidity) were excluded for the following analysis: *job adaptations* and *pre-operative type of employment.*

Successful RTW was defined as any resumption of work after shoulder arthroplasty, including return to either the previous or a modified job, on either a full-time or part-time basis. For analysis, patients were stratified into 2 groups based on their work status at follow-up: those who successfully returned to work (RTW group) and those who did not (no RTW group). Patient demographics, PROMs, and responses to the work-specific questionnaire were compared between the 2 groups.

### Statistical analysis

An a priori sample size calculation was performed based on data from literature.^19^ With power set at 90%, a significance level of 0.05, and a margin of error of ±10%, the required minimum sample size was calculated to be 90 patients.

The Shapiro-Wilk test was used to check for normal distribution of the obtained data. Depending on the normality of the data, continuous data were presented with either mean and standard deviation or median and range, and either the Student's *t*-test or the Mann–Whitney *U* test was used to test for differences in continuous data between groups. Categorical variables were presented as counts and percentages and compared using the chi-squared test. A multivariable logistic regression analysis was performed to identify factors associated with successful RTW. Covariates were selected based on demographic relevance, clinical relevance, plausible association with RTW, and potential relevance for pre-operative counseling, and/or post-operative work-reintegration strategies. To reduce the risk of overfitting, the number of covariates was limited. Job physicality was dichotomized as high/very high demand vs. very low/low/neutral demand, pre-operative sick leave as >1 year vs. ≤1 year, and post-operative support received at work as any support vs. no support. Results are reported as odds ratios (ORs) with 95% confidence intervals (CIs). A *P* value <.05 was considered statistically significant.

## Results

### Patient demographics

A total of 280 patients younger than 63 years at the time of surgery underwent a shoulder arthroplasty procedure. Of these, 133 patients were excluded for the following reasons: 55 were on long-term disability for nonshoulder-related reasons, 45 had taken early retirement and were therefore retired at the time of surgery, 22 had died during follow-up, and 11 were excluded because of limited understanding of Dutch. The remaining 147 patients met the inclusion criteria and were subsequently invited to participate. With a response rate of 70%, 103 patients were ultimately included in the study.

The final cohort consisted of 41 females and 62 males with a mean age at the time of surgery of 52.2 ± 7.8 years (Table I). The mean BMI was 28.8 ± 4.9 and the mean ASA score was 2.0 ± 0.6. The dominant limb was involved in 68% of the patients. The mean follow-up duration was 74.3 ± 39.1 months. Eleven patients were pre-operatively on long-term disability (“invalidity”). In all these cases, the disability status was directly related to the patient's shoulder pathology.

Within the cohort, 53 patients (51%) underwent aTSA, 44 patients (43%) rTSA, and 6 patients (6%) Humeral hemiarthroplasty (HHA). The primary surgical indications were glenohumeral osteoarthritis (54%), cuff tear arthropathy (15%), proximal humerus fracture (10%), revision surgery (14%), and other causes (8%) like avascular necrosis of the humeral head and postinfectious arthropathy.

### Return to work

**Figure 1:**
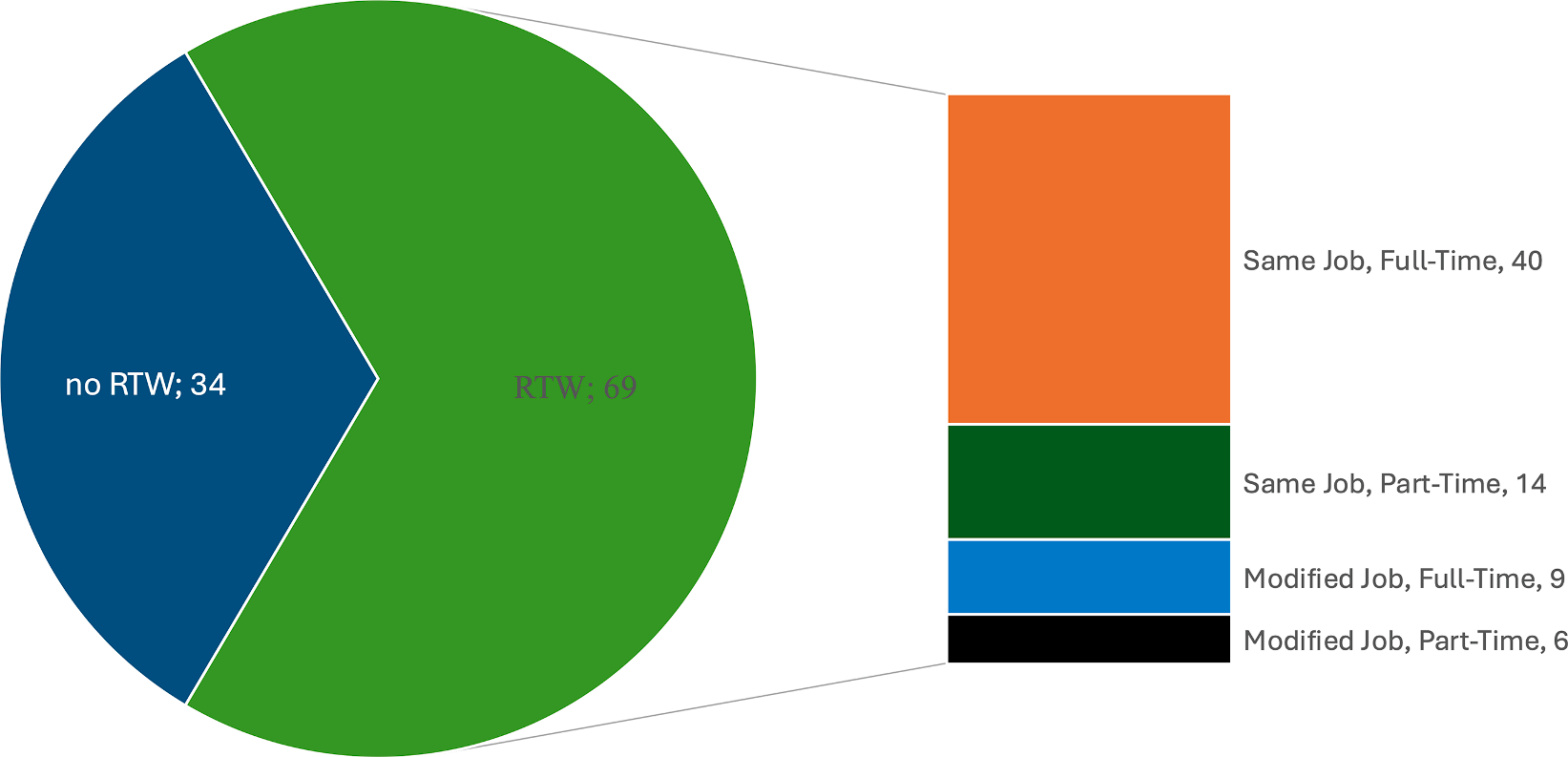
Graphic overview of return-to-work outcomes categorized by job function (same vs. modified) and intensity (full-time vs. part-time).

Sixty-nine patients (67%) successfully returned to work, of whom 40 patients (58%) resumed their previous job on a full-time basis (Fig. 1). The mean time to return to work was 16.2 ± 13.2 weeks. Table I summarizes the variables included in the statistical analysis, with mean values or frequencies depicted for the RTW and no RTW groups, with the associated *P* values. Age at surgery, dominant side, gender, BMI, and ASA score had no significant impact on the RTW rate. Among social factors, only the educational level was significantly associated with RTW outcomes. Smoking status and marital status did not interfere with RTW.

**Figure 2:**
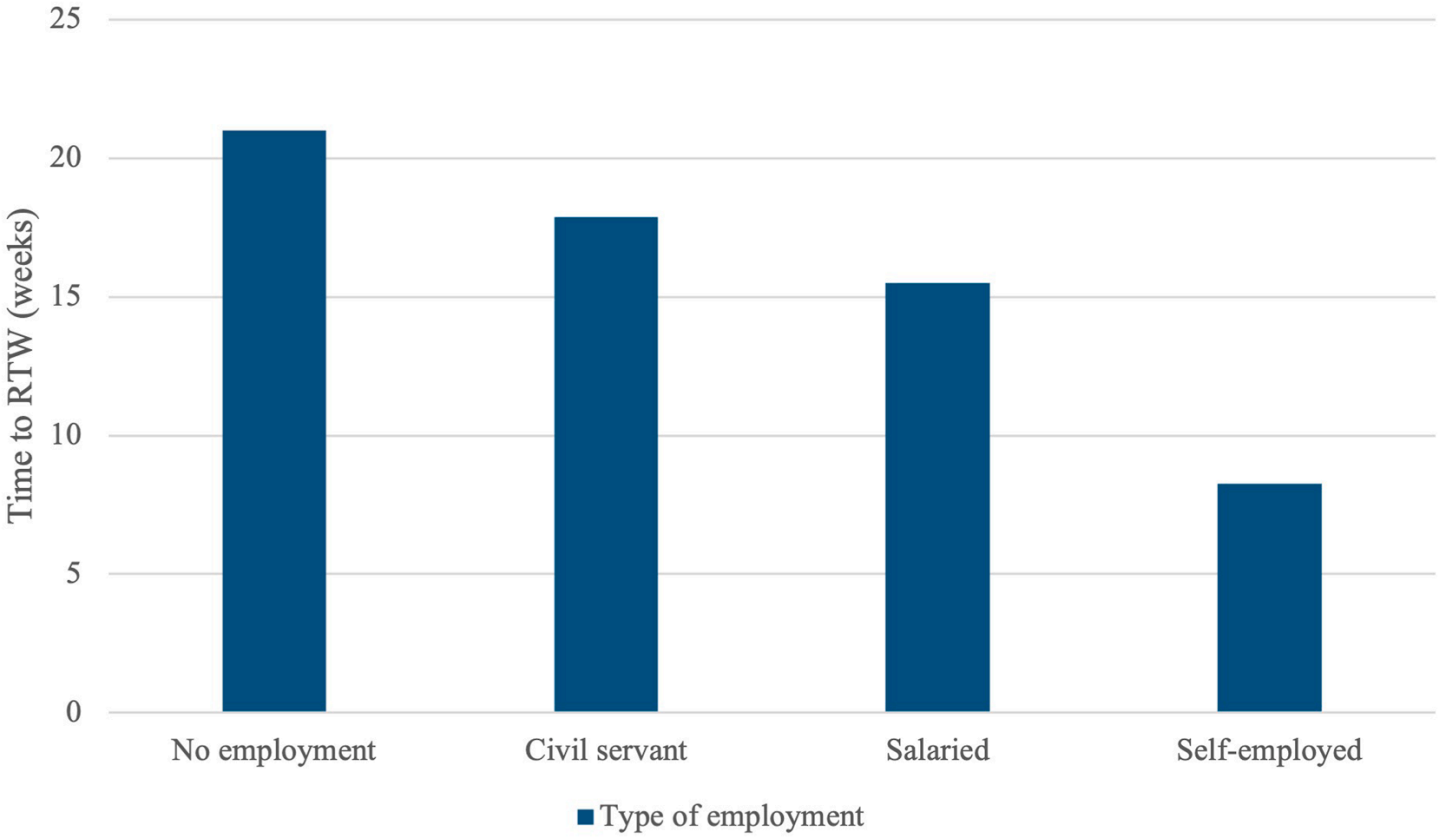
Graphic overview of the differences in time to return-to-work (weeks) by type of employment.

Work-related factors had a notable influence: patients engaged in physically demanding occupations and those who reported insufficient post-operative workplace support were less likely to return to work. Similarly, patients who had been on pre-operative sick leave for more than one year demonstrated lower RTW rates. On the other hand, type of employment and pre-operative satisfaction at work did not significantly impact RTW. While the type of employment did not affect whether patients returned to work, it did have a significant impact on the time to RTW (Fig. 2). Self-employed individuals returned to work significantly earlier (8 weeks) compared to salaried employees (15 weeks), civil servants (18 weeks), and unemployed individuals (21 weeks) at the time of surgery.

RTW rates did not differ significantly between the different types of arthroplasties, with 70% for aTSA, 61% for rTSA, and 83% for HHA. Similarly, the surgical indication, presence of complications, and need for revision surgery did not impact RTW.

In the multivariable logistic regression analysis, high or very high physically demanding work remained associated with lower odds of successful RTW (OR, 0.04; 95% CI, 0.004–0.32; *P* = .003). Pre-operative sick leave exceeding one year was also associated with lower odds of successful RTW (OR, 0.14; 95% CI, 0.03–0.66; *P* = .013). Post-operative support received at work was not significantly associated with successful RTW after adjustment (OR, 2.39; 95% CI, 0.68–8.44; *P* = .176). Age was not associated with successful RTW (OR, 1.00; 95% CI, 0.92–1.08; *P* = .951).

### Patient-reported outcome measures (PROMs)

All the PROMs (including Subjective Shoulder Value, Oxford Shoulder Score, Simple Shoulder Test score, and Work-Related Questionnaire for Upper extremity disorders score) were statistically significantly higher in the RTW group compared to the group of patients who failed to return to work.

## Discussion

In this study, we investigated the RTW rates and its associated factors following shoulder arthroplasty. Compared to knee and hip arthroplasty, RTW following shoulder arthroplasty remains less extensively studied. Overall, 67% of shoulder arthroplasty patients were able to RTW, although only 58% of patients of this subgroup were able to resume their previous job on a full-time basis. This overall rate is comparable with RTW rates reported after other major joint replacements. Pooled data from recent systematic reviews have reported RTW rates of 65% to 70% following hip and knee arthroplasty; however, more recent studies indicate a trend toward higher rates over time, reaching up to 90% in some cohorts.^15^^,^^18^

Reported RTW rates for shoulder arthroplasty in the existing literature vary widely, ranging from 56% to 92%.^11^^,^^19^ Interestingly, the studies reporting RTW rates exceeding 90% often included only patients who underwent aTSA. In contrast, only 70% of aTSA patients in our cohort returned to work. This discrepancy may be attributed to the differences in study populations, as previous studies primarily included younger individuals (<55 years) and selected participants who explicitly intended to return to occupational and/or sporting activities.^6^^,^^13^

The mean time to RTW in our study (16 weeks) is at the higher end of the range reported in previous literature (1–4 months).^11^ This prolonged time to RTW was particularly evident among civil servants and salaried employees, whose time to RTW was significantly longer than those of self-employed individuals. A potential contributing factor to this discrepancy might be the structure of the Belgian social security system. Unlike self-employed individuals, civil servants and salaried employees can benefit from a more extensive social safety net, which facilitates an extended period of guaranteed income during medical leave. This may partly explain the longer time to RTW observed in these groups.

Another important finding of this study is that all PROMs were significantly better in the group of patients that successfully returned to work compared to those who did not. These results support the idea that higher functional recovery and greater patient satisfaction contribute to the ability to return to work. Similar findings have been reported in previous studies on different surgical procedures, where superior clinical outcomes correlated with higher RTW rates.^12^^,^^16^ This underscores the value of PROMs not only as indicators for surgical success but also as a predictor for successful RTW.

The most significant predictive factors for unsuccessful RTW in our study were lower educational level, high physical job demands, pre-operative sick leave exceeding one year, and limited post-operative support received at work. In the exploratory multivariable logistic regression analysis, high or very high physically demanding work and pre-operative sick leave exceeding one year remained associated with lower odds of successful RTW, whereas post-operative support received at work was not significantly associated with RTW after adjustment. The association between educational level and RTW likely reflects job type, as individuals with higher education levels (college/university) are more likely employed in less physically demanding jobs.^5^ This trend has also been observed in studies investigating RTW after other major joint replacements, where patients who are employed in physically high-demanding jobs had significantly lower RTW rates compared to those who are employed in more sedentary jobs.^8^^,^^21^ Similarly, pre-operative sick leave longer than one year has also been identified as a predictive factor for poor RTW rates after total knee arthroplasty.^22^ Nevertheless, in our cohort, 6 of the 23 patients with pre-operative sick exceeding one year successfully returned to work, suggesting that in selected cases, shoulder arthroplasty can have a positive impact on socioeconomic reintegration. Contrary to expectations, pre-operative job satisfaction was not associated with RTW outcomes. This contrasts with previous research showing that a strong pre-operative intent to return to work can predict successful RTW after total joint arthroplasty.^1^

Other patient-related and social factors—such as age at time of surgery, side affected, gender, BMI, ASA score, smoking status, and marital status—did not significantly impact the RTW rate. Similarly, surgical factors such as the type of arthroplasty (aTSA, rTSA, HHA) performed, surgical indication (osteoarthritis, cuff tear arthropathy, trauma), the occurrence of post-operative complications, or the need for revision surgery were not associated with differences in RTW outcomes. These findings are consistent with prior studies on shoulder arthroplasty, which also report minimal to no impact of demographic or surgical factors on RTW rates.^8^^,^^19^ Similar patterns have been observed in hip and knee arthroplasty cohorts, where demographic and surgical variables also showed limited predictive value for RTW, while work-related variables such as job physicality and educational level significantly impacted RTW rates.^20^^,^^21^

Our findings may provide relevant insights for healthcare policy. While patient- and surgery-related factors appear to have little influence on RTW outcomes, social and work-related factors—such as educational level, job physicality, pre-operative sick leave, and post-operative workplace support—appear to be associated with RTW status after shoulder arthroplasty. These insights highlight several potential opportunities for governmental action. Future studies should therefore investigate whether workplace reintegration programs, targeted support for patients with prolonged pre-operative sick leave, and re-education programs for patients with physically demanding jobs can improve RTW after shoulder arthroplasty.

The added value of this study in addition to current literature lies in its comprehensive assessment of patient-related, social, work-related, and surgical variables in a large cohort. Nevertheless, this study has several limitations. First, its retrospective design introduces the potential for selection bias. Second, as a single-center study conducted in Belgium, the findings of the study may not be entirely generalizable to healthcare systems or work environments in other countries due to socioeconomic differences. Third, the exclusion of patients with limited understanding of Dutch may have introduced some selection bias, particularly in relation to the observed association between educational level and RTW. However, the size of this subgroup was small, and therefore the potential impact on the overall findings is likely limited. Finally, the multivariable analysis should be considered exploratory because of the cohort size and the limited number of patients who did not return to work.

## Conclusion

Approximately two-thirds of patients successfully returned to work after shoulder arthroplasty. Predictive factors for successful RTW included educational level, job physicality, absence of prolonged pre-operative sick leave and the amount of post-operative support received at work. The type of arthroplasty, indication, presence of complications and need for revision surgery did not significantly impact RTW. These findings may guide pre-operative counseling and help manage post-operative patient expectations in individuals of working age undergoing shoulder arthroplasty. Furthermore, they may help identify patients who could benefit from targeted programs to improve post-operative socioeconomic (re)integration.

## Disclaimers:

Funding: Willem Geuskens is supported by the RCSI StAR PhD Programme. The funder had no role in the study design, data collection, data analysis, interpretation of the findings, or decision to submit the manuscript for publication.

Conflicts of interest: The Institute for Orthopaedic Research and Training (IORT), Department of Development and Regeneration, KU Leuven, Division of Orthopaedics, University Hospitals Leuven reports research grant funding from the following organizations, which are not related to the topic of this work: charitable donations from Zimmer Biomet (EDW-ZIMMER-O2030), the PROSPEROS I (2014TC16RFCB046) and PROSPEROS II project (ZL3C150100-401) funded by the Interreg VA Flanders–The Netherlands program. None of these outside sources or funders were involved in the study design, data collection, data analysis, or the preparation or editing of the manuscript. Filip Verhaegen reports consultancy agreements with Zimmer Biomet and Exactech INC. Philippe Debeer reports consultancy agreement with Materialise. Willem-Jan Metsemakers reports consultancy agreements with Johnson & Johnson MedTech and Zimmer Biomet. Filip Verhaegen and Philippe Debeer hold Hip and Shoulder Research Johnson educational chair. These relationships have no bias on this work and did not inappropriately influence this study. Any additional authors, their immediate families, and any research foundations with which they are affiliated have not received any financial payments or other benefits from any commercial entity related to the subject of this article.
